# Body weight history from young adulthood and subsequent blood pressure trajectories in a Japanese employment-based health-check cohort: a retrospective longitudinal study

**DOI:** 10.1186/s12889-026-29097-z

**Published:** 2026-08-19

**Authors:** Katsumi Iizuka

**Affiliations:** Department of Clinical Nutrition, Fujita Health University, 1-98, Dengakugakubo, Kutsukakecho, Toyoake, 470-1192 Japan

**Keywords:** Body weight history, Blood pressure trajectories, Obesity, Underweight, Longitudinal studies, Young adults

## Abstract

**Background:**

Whether long-term body weight history across adulthood, rather than body weight at a single time point, is associated with later blood pressure (BP) trajectories remains uncertain. We examined this association in a large longitudinal health checkup cohort.

**Methods:**

We analyzed repeated systolic and diastolic blood pressure measurements from 406,814 participants in a Japanese employment-based health-check cohort using sex-stratified linear mixed-effects models with age-by-group interactions and adjustment for sleep, exercise habits, physical activity, and walking speed. Participants were classified into three groups: history of underweight (BMI < 18.5 kg/m² at least once; *n* = 80,890), normal weight only (BMI 18.5 to < 25.0 kg/m² throughout the 20s; *n* = 228,343), and history of obesity (BMI ≥ 25.0 kg/m² at least once; *n* = 97,581). Sensitivity analyses among 402,651 participants additionally adjusted for eating speed, breakfast skipping, and late-evening meals. The underweight-history and obesity-history groups were further subdivided by the proportion of follow-up time spent underweight or obese (0–25%, 25–50%, 50–75%, and 75–100%).

**Results:**

Participants with a history of obesity during their 20s showed higher BP trajectories than those with normal weight only, and these differences increased with age. In contrast, participants with a history of underweight showed lower BP trajectories than the normal-weight-only group. Age-by-group interactions were significant in women and men for both systolic and diastolic BP (all *P* < 0.001). Additional adjustment for eating behaviors had minimal influence on these associations. In the nine-group analysis, the groups with 50–75% and 75–100% of follow-up time spent obese showed higher BP trajectories than the normal-weight-only group, whereas the groups with 50–75% and 75–100% of follow-up time spent underweight showed lower BP trajectories.

**Conclusions:**

These findings suggest that shorter durations of obesity and underweight may be associated with BP trajectories closer to those of the normal-weight-only group. Overall, long-term body weight history may be more informative than body weight at a single time point when evaluating later BP burden.

**Supplementary Information:**

The online version contains supplementary material available at https://doi.org/10.1186/s12889-026-29097-z.

## Background

Hypertension is influenced by both genetic predispositions and environmental factors; however, the factors that are currently modifiable are predominantly environmental [1, 2]. Among these factors, weight gain is a well-established risk factor for hypertension, and increasing evidence suggests that not only the degree of obesity, but also its duration and cumulative exposure, contribute to elevated blood pressure and cardiovascular risk [3–6]. Dietary habits and eating behaviors may also play a role in the development of hypertension [7–11]. For example, skipping breakfast and fast eating have been reported to be associated with a higher risk of hypertension [7–9].

Although the associations of obesity history with hypertension risk have been documented in many studies, evidence remains limited regarding how body weight history in young adulthood, including both obesity and underweight, is related to subsequent blood pressure trajectories over the long term and whether these associations are influenced by eating behaviors. In particular, few studies have evaluated eating behaviors within the broader framework of body weight history rather than body weight at a single time point [7–9].

Therefore, in the present study, we first examined the association between body weight history during the 20s and subsequent blood pressure trajectories. We then performed sensitivity analyses to assess the influence of baseline eating behaviors. Furthermore, we examined whether, among individuals with a history of underweight or obesity during their 20s, a greater proportion of subsequent follow-up time spent underweight or obese was associated with distinct blood pressure trajectories. Through these analyses, we aimed to clarify the importance of focusing on body weight history, rather than body weight at a single time point, when evaluating subsequent blood pressure trajectories.

## Methods

### Study population and definition of weight-history groups

**Fig. 1:**
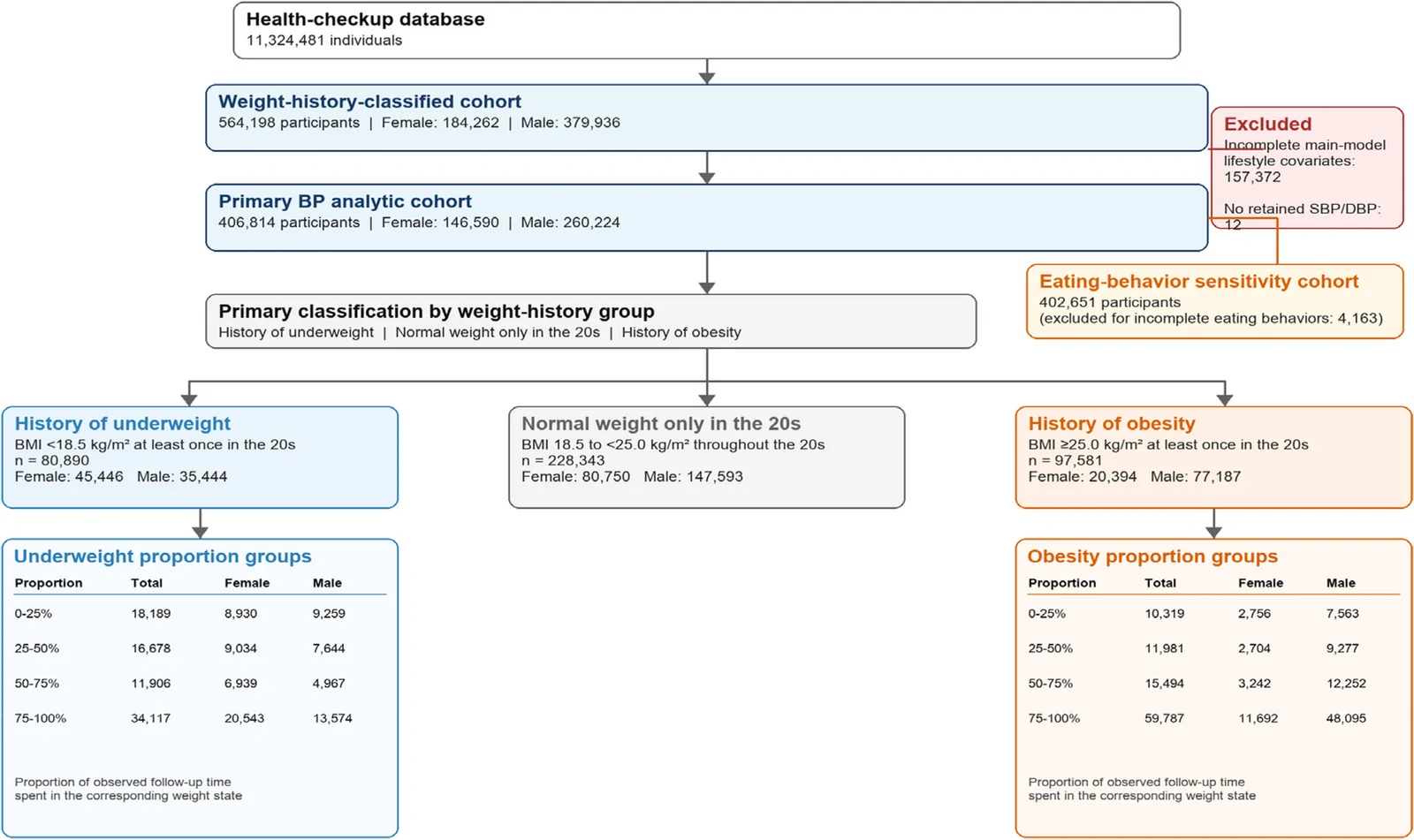
Cohort flow and nine-group weight-history classification. The weight-history-classified cohort comprised participants whose health checkup follow-up began during their 20s and who had at least five health checkup records. After excluding 157,372 participants with incomplete main-model lifestyle covariates and 12 without retained SBP or DBP measurements, 406,814 participants comprised the primary analytic cohort. The primary cohort was classified into three mutually exclusive groups: history of underweight, normal weight only throughout the 20s, and history of obesity. The underweight-history and obesity-history groups were further subdivided according to the proportion of observed follow-up time spent underweight or obese (0–25%, 25–50%, 50–75%, and 75–100%), yielding nine groups. Participants with underweight history were assigned first; the normal-weight-only group included participants with neither underweight nor obesity during their 20s; and the obesity-history group excluded participants already assigned to the former groups. For the eating-behavior sensitivity analysis, 4,163 participants with incomplete eating-behavior data were additionally excluded, leaving 402,651 participants. BP, blood pressure; DBP, diastolic blood pressure; SBP, systolic blood pressure

This retrospective longitudinal cohort study used health checkup data obtained from JMDC Inc. (Tokyo, Japan), covering Japanese employment-based health insurance beneficiaries between 2005 and 2025. The database was not restricted to healthcare professionals; it included employees from multiple employment sectors and their dependents. Accordingly, the source population should be regarded as an employment-based insured population rather than a representative sample of the general Japanese population. During this period, 11,324,481 individuals, including 5,004,417 women and 6,320,064 men, were registered in the database. The underlying longitudinal weight-history cohort included participants whose health checkup follow-up began between ages 20 and 29 years and who had at least five health checkup records, yielding 564,198 participants (184,262 women and 379,936 men) [12]. Health checkup records were generally available at approximately annual intervals, although their exact number and timing varied among participants. The JMDC health checkup records classified participants as male or female, and information on gender identity was unavailable; therefore, sex rather than gender was used in the present study (Fig. 1).

For the present BP analysis, we used this underlying cohort of 564,198 participants with classifiable weight history. Of these, 157,372 were excluded because at least one main-model lifestyle covariate (sleep, exercise habits, physical activity, or walking speed) was unavailable, and 12 were excluded because no SBP or DBP measurement was retained for analysis. The primary analytic cohort therefore comprised 406,814 participants (146,590 women and 260,224 men). The sensitivity analysis additionally required complete baseline information on eating speed, breakfast skipping, and late-evening meals and included 402,651 participants (145,191 women and 257,460 men). BP was measured by nurses during routine health checkups; however, the database did not specify whether measurements were obtained manually or with automated devices and did not provide detailed information on measurement protocols or conditions. No additional exclusions were applied on the basis of baseline BP, a history of hypertension, or antihypertensive medication use.

Weight-history groups were defined using BMI records obtained during participants’ 20s. In the primary analysis, participants were classified into three mutually exclusive categories: history of underweight (BMI < 18.5 kg/m² at least once), normal weight only throughout the 20s, and history of obesity (BMI ≥ 25.0 kg/m² at least once) [12–14]. To ensure mutual exclusivity, participants with any history of underweight were assigned first, followed by those who remained at normal weight throughout their 20s, and finally those with a history of obesity after exclusion of the former two groups.

For the secondary detailed analysis, the underweight-history and obesity-history groups were further subdivided according to the proportion of observed follow-up time spent in the respective weight states (0–25%, 25–50%, 50–75%, and 75–100%). Together with the normal-weight-only group, this approach yielded nine mutually exclusive weight-history groups.

### Assessment of eating behaviors and covariates

Lifestyle and eating-behavior information was obtained from standardized questionnaire items completed by participants as part of routine health-check practice. The investigators did not administer the questionnaires retrospectively; responses recorded by health-check providers were included in the JMDC database and analyzed as secondary data. Eating speed was self-classified as fast, normal, or slow. Breakfast skipping was defined according to the corresponding questionnaire item, and a late-evening meal was defined as eating dinner within 2 h before bedtime at least three times per week [12]. These were standardized single-item questions routinely used in Japanese health checkups rather than independently validated multi-item questionnaires. Information on whether the questionnaire was completed on paper or electronically, its precise timing relative to the examination, and whether assistance was provided was unavailable in the database.

Baseline covariates were defined using responses from the first available health checkup between ages 20 and 29 years. Sleep adequacy was assessed by a yes/no response to the question, “Do you get adequate rest from sleep?” Exercise habits were defined as exercise causing light sweating for at least 30 min per session, at least twice weekly, for at least 1 year. Physical activity was defined as walking or equivalent activity for at least 1 h/day [12]. Walking speed was defined as self-reported walking speed faster than that of same-sex individuals of a similar age [12]. All questionnaire-based variables were self-reported and were therefore potentially subject to reporting bias and misclassification.

### Statistical analysis

Because this was a retrospective database study, no a priori sample-size calculation was performed. All eligible participants were included to maximize the precision of age-specific trajectory estimates and to support sex-stratified and nine-group analyses. Given the large sample size, findings were interpreted using age-specific estimated marginal means and adjusted mean differences in mmHg with their 95% confidence intervals, rather than from P values alone.

All analyses were performed separately for women and men. Repeated SBP and DBP measurements were analyzed using linear mixed-effects models. In the primary analysis, participants were classified into three major weight-history groups: history of underweight, normal weight only throughout the 20s, and history of obesity. To evaluate whether age-related SBP and DBP trajectories differed according to weight-history group, the models included weight-history group, age, and their interaction. Age was modeled using a natural spline with three degrees of freedom. Sleep adequacy, exercise habits, physical activity, and walking speed were included a priori as adjustment covariates because of their potential associations with both body-weight history and BP; they were not prespecified as primary exposures or effect modifiers. Participant-specific random intercepts were included to account for within-person correlation due to repeated measurements.

As a sensitivity analysis, the three-group model was additionally adjusted for baseline eating behavior variables, including eating speed, breakfast skipping, and late-evening meal consumption. Baseline eating behaviors were used to preserve temporal ordering and to avoid adjustment for behaviors that may have changed in response to subsequent weight gain or BP elevation. Estimated marginal means at ages 25, 35, and 45 years were compared with those from the primary model. Age-by-group interactions were evaluated using likelihood ratio tests comparing models with and without interaction terms. To summarize group-specific trajectories, estimated marginal means and 95% confidence intervals for SBP and DBP were calculated at ages 25, 30, 35, 40, and 45 years.

Because significant age-by-group interactions were observed in the three-group analysis, a more detailed nine-group analysis was subsequently conducted. In this analysis, the underweight-history and obesity-history groups were further subdivided according to the proportion of the subsequent follow-up period spent underweight or obese (0–25%, 25–50%, 50–75%, and 75–100%), whereas the normal-weight-only group was retained as a single reference category. The same modeling strategy was then applied to the nine-group classification. For both the three-group and nine-group classifications, age-specific contrasts between each underweight- or obesity-history group and the normal-weight-only group were expressed as adjusted mean differences in mmHg with 95% confidence intervals.

All statistical tests were two-sided, and *P* < 0.05 was considered statistically significant. All analyses were performed using R version 4.4.1 (R Foundation for Statistical Computing, Vienna, Austria). The main packages used were data.table, lme4, emmeans, splines, and ggplot2.

### Ethics

All data provided by JMDC Inc. were fully deidentified before analysis. As the dataset was anonymized, individual optouts were not feasible. This was disclosed through publicly available information in accordance with institutional requirements. The study protocol was approved by the Institutional Review Board of Fujita Health University (Approval No. HM25-712, April 14, 2026; and Approval No. HM26-180, June 23, 2026), and the study was conducted in accordance with the Declaration of Helsinki.

## Results

### Participant characteristics

The primary analytic cohort included 80,890 participants with a history of underweight during their 20s (45,446 women and 35,444 men), 228,343 who maintained normal weight throughout their 20s (80,750 women and 147,593 men), and 97,581 with a history of obesity (20,394 women and 77,187 men) (Table 1). Mean baseline age ranged from 24.2 to 24.9 years in women and from 23.7 to 24.9 years in men, while the median observed age span was 6 years across groups. Fast eating was least common in the underweight-history group (women: 17.5% [7,910]; men: 19.6% [6,908]) and most common in the obesity-history group (women: 29.5% [5,972]; men: 48.1% [36,907]). Conversely, slow eating was most common in the underweight-history group (women: 24.9% [11,232]; men: 21.5% [7,594]) and least common in the obesity-history group (women: 13.2% [2,673]; men: 6.1% [4,683]). Eating behaviors varied less across groups in men than in women (Table 1).

**Table 1:** Baseline characteristics of the primary analytic cohort by weight-history group

Variable	History of UW	NW only in 20s	History of Ob
Participants, n	F: 45,446 M: 35,444	F: 80,750 M: 147,593	F: 20,394 M: 77,187
Age at baseline, mean (SD), years	F: 24.2 (2.7) M: 23.7 (2.8)	F: 24.9 (2.8) M: 24.9 (2.9)	F: 24.4 (2.8) M: 24.5 (2.9)
BMI at baseline, mean (SD), kg/m²	F: 18.0 (1.2) M: 17.9 (1.1)	F: 20.9 (1.5) M: 21.3 (1.5)	F: 26.6 (3.9) M: 26.7 (3.7)
Waist circumference at baseline, mean (SD), cm	F: 66.0 (4.4) M: 67.5 (3.9)	F: 71.9 (5.4) M: 75.7 (5.3)	F: 84.6 (10.0) M: 89.2 (10.0)
Observed age span, median [Q1, Q3], years	F: 6.0 [5.0, 7.0] M: 6.0 [5.0, 8.0]	F: 6.0 [5.0, 7.0] M: 6.0 [5.0, 8.0]	F: 6.0 [5.0, 7.0] M: 6.0 [5.0, 8.0]
Total health-check visits, median [Q1, Q3]	F: 6.0 [5.0, 8.0] M: 7.0 [6.0, 9.0]	F: 6.0 [5.0, 8.0] M: 7.0 [6.0, 9.0]	F: 7.0 [6.0, 8.0] M: 7.0 [6.0, 9.0]
Sleep: adequate/restful, % (n)	F: 67.3% (30602) M: 66.1% (23418)	F: 66.7% (53883) M: 67.0% (98828)	F: 62.2% (12686) M: 65.4% (50463)
Exercise habit: yes, % (n)	F: 7.2% (3279) M: 16.0% (5666)	F: 10.4% (8430) M: 23.8% (35147)	F: 11.3% (2305) M: 24.5% (18916)
Physical activity/walking: yes, % (n)	F: 46.1% (20958) M: 54.9% (19457)	F: 48.7% (39346) M: 56.4% (83299)	F: 48.8% (9962) M: 55.3% (42695)
Walking speed: fast, % (n)	F: 38.3% (17421) M: 48.7% (17272)	F: 39.1% (31553) M: 48.6% (71775)	F: 33.6% (6844) M: 43.1% (33236)
Eating speed: fast, % (n)	F: 17.5% (7910) M: 19.6% (6908)	F: 24.0% (19226) M: 32.8% (48116)	F: 29.5% (5972) M: 48.1% (36907)
Eating speed: slow, % (n)	F: 24.9% (11232) M: 21.5% (7594)	F: 17.1% (13758) M: 11.5% (16878)	F: 13.2% (2673) M: 6.1% (4683)
Breakfast skipping: yes, % (n)	F: 28.5% (12933) M: 39.5% (13960)	F: 29.8% (24017) M: 37.4% (54977)	F: 36.1% (7332) M: 39.7% (30547)
Late-evening meal: yes, % (n)	F: 33.5% (15181) M: 39.7% (14049)	F: 34.2% (27611) M: 41.0% (60374)	F: 36.4% (7415) M: 41.6% (32022)

Values are shown as mean (SD), median [interquartile range], or % (n), unless otherwise indicated. Percentages for eating behaviors were calculated among participants with available responses. *F* female, *M* male, *UW* underweight, *NW* normal weight only throughout the 20s, *Ob* obesity

Sleep adequacy and physical activity varied within relatively narrow ranges across the weight-history groups. Adequate or restful sleep ranged from 62.2% to 67.3% in women and from 65.4% to 67.0% in men, while physical activity ranged from 46.1% to 48.8% and from 54.9% to 56.4%, respectively. Regular exercise was less common in the underweight-history group (women, 7.2%; men, 16.0%) than in the normal-weight-only group (women, 10.4%; men, 23.8%) or obesity-history group (women, 11.3%; men, 24.5%). Fast walking was somewhat less common in the obesity-history group (women, 33.6%; men, 43.1%) than in the other weight-history groups (Table 1).

### Associations of systolic and diastolic blood pressure trajectories with the three-group weight-history classification and sensitivity analyses with baseline eating behaviors

**Fig. 2:**
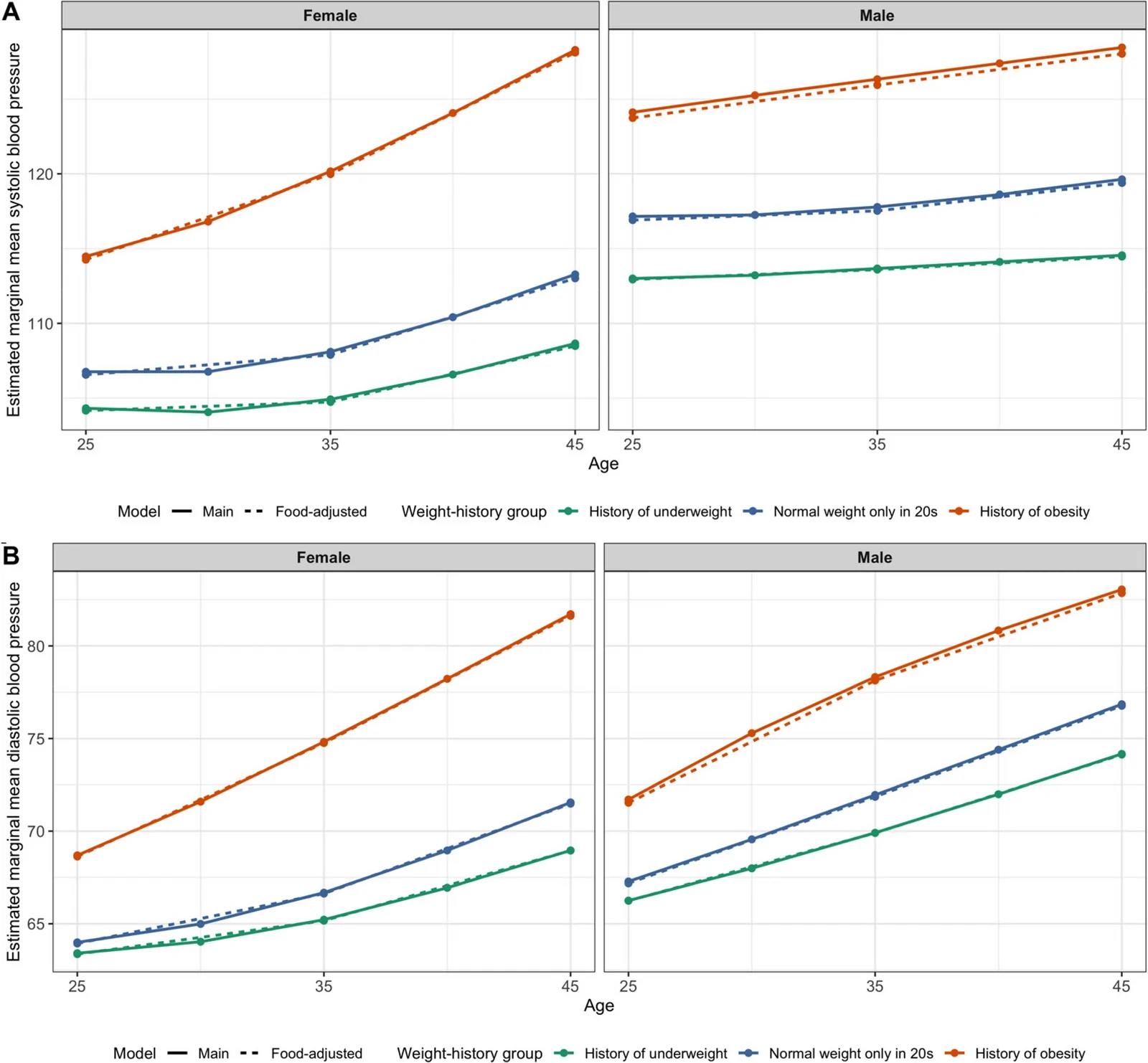
Three-group blood pressure trajectories and sensitivity analyses. Estimated marginal mean systolic blood pressure (SBP; panel **A**) and diastolic blood pressure (DBP; panel **B**) trajectories derived from sex-stratified linear mixed-effects models are shown for the three major weight-history groups: history of underweight, normal weight only throughout the 20s, and history of obesity. In each panel, results are shown separately for women and men. Solid lines indicate the main model adjusted for sleep, exercise habits, physical activity, and walking speed, and dashed lines indicate the model additionally adjusted for baseline eating behaviors, including eating speed, breakfast skipping, and late-evening meal consumption. Error bars indicate 95% confidence intervals. Age-by-group interaction results are presented in Table 2

Likelihood ratio tests showed significant age-by-group interactions in the three-group models for both women and men (all *P* < 0.001), indicating that age-related changes in SBP and DBP differed according to weight-history group. Therefore, we examined SBP and DBP trajectories across the three major weight-history groups. In both women and men, participants with a history of obesity showed higher SBP and DBP trajectories than those with a history of underweight or those who maintained normal weight throughout their 20s, whereas participants with a history of underweight tended to have lower trajectories. Consistent with the significant interaction terms, the separation among groups became more apparent with increasing age (Fig. 2 and Supplementary Table 1). At age 45 years, compared with the normal-weight-only group, the adjusted SBP/DBP differences in the underweight-history group were − 4.6/-2.6 mmHg in women and − 5.1/-2.7 mmHg in men, whereas those in the obesity-history group were + 15.0/+10.2 mmHg in women and + 8.8/+6.2 mmHg in men (Supplementary Table 3).

**Table 2:** Estimated blood pressure trajectories at ages 25, 35, and 45 years

Outcome	Sex	Weight-history group	Age 25 years	Age 35 years	Age 45 years
SBP	Female	History of UW	104.164 [104.044, 104.283]	104.741 [104.600, 104.883]	108.480 [108.002, 108.958]
SBP	Female	NW only in 20s	106.555 [106.454, 106.657]	107.891 [107.781, 108.002]	113.009 [112.683, 113.335]
SBP	Female	History of Ob	114.258 [114.103, 114.412]	119.980 [119.788, 120.171]	128.114 [127.341, 128.886]
SBP	Male	History of UW	112.932 [112.820, 113.044]	113.592 [113.447, 113.736]	114.464 [113.861, 115.067]
SBP	Male	NW only in 20s	116.908 [116.842, 116.975]	117.529 [117.458, 117.601]	119.387 [119.153, 119.621]
SBP	Male	History of Ob	123.739 [123.655, 123.823]	125.936 [125.838, 126.035]	128.033 [127.640, 128.426]
DBP	Female	History of UW	63.360 [63.267, 63.454]	65.166 [65.056, 65.277]	68.952 [68.581, 69.322]
DBP	Female	NW only in 20s	63.933 [63.854, 64.013]	66.629 [66.543, 66.716]	71.485 [71.232, 71.738]
DBP	Female	History of Ob	68.628 [68.507, 68.749]	74.758 [74.609, 74.907]	81.624 [81.025, 82.223]
DBP	Male	History of UW	66.244 [66.156, 66.332]	69.900 [69.788, 70.012]	74.134 [73.680, 74.588]
DBP	Male	NW only in 20s	67.183 [67.131, 67.235]	71.856 [71.800, 71.912]	76.768 [76.592, 76.945]
DBP	Male	History of Ob	71.534 [71.468, 71.600]	78.131 [78.055, 78.208]	82.855 [82.559, 83.151]

Values are shown as mean [95% CI]. Additionally adjusted models included eating speed, breakfast skipping, and late-evening meal consumption. *CI* confidence interval, *SBP* systolic blood pressure, *DBP* diastolic blood pressure, *UW* underweight, *NW* normal weight only throughout the 20s, *Ob* obesity

Next, we performed sensitivity analyses by additionally adjusting the three-group models for baseline eating behaviors, including eating speed, breakfast skipping, and late-evening meal consumption. The estimated marginal means before and after this additional adjustment were highly similar for both SBP and DBP, and the overall trajectory patterns were essentially unchanged (Fig. 2; Table 2). These findings indicate that baseline eating behaviors had minimal influence on the association between weight-history group and blood pressure trajectories.

### Associations of systolic and diastolic blood pressure trajectories with the nine-group weight-history classification

**Fig. 3:**
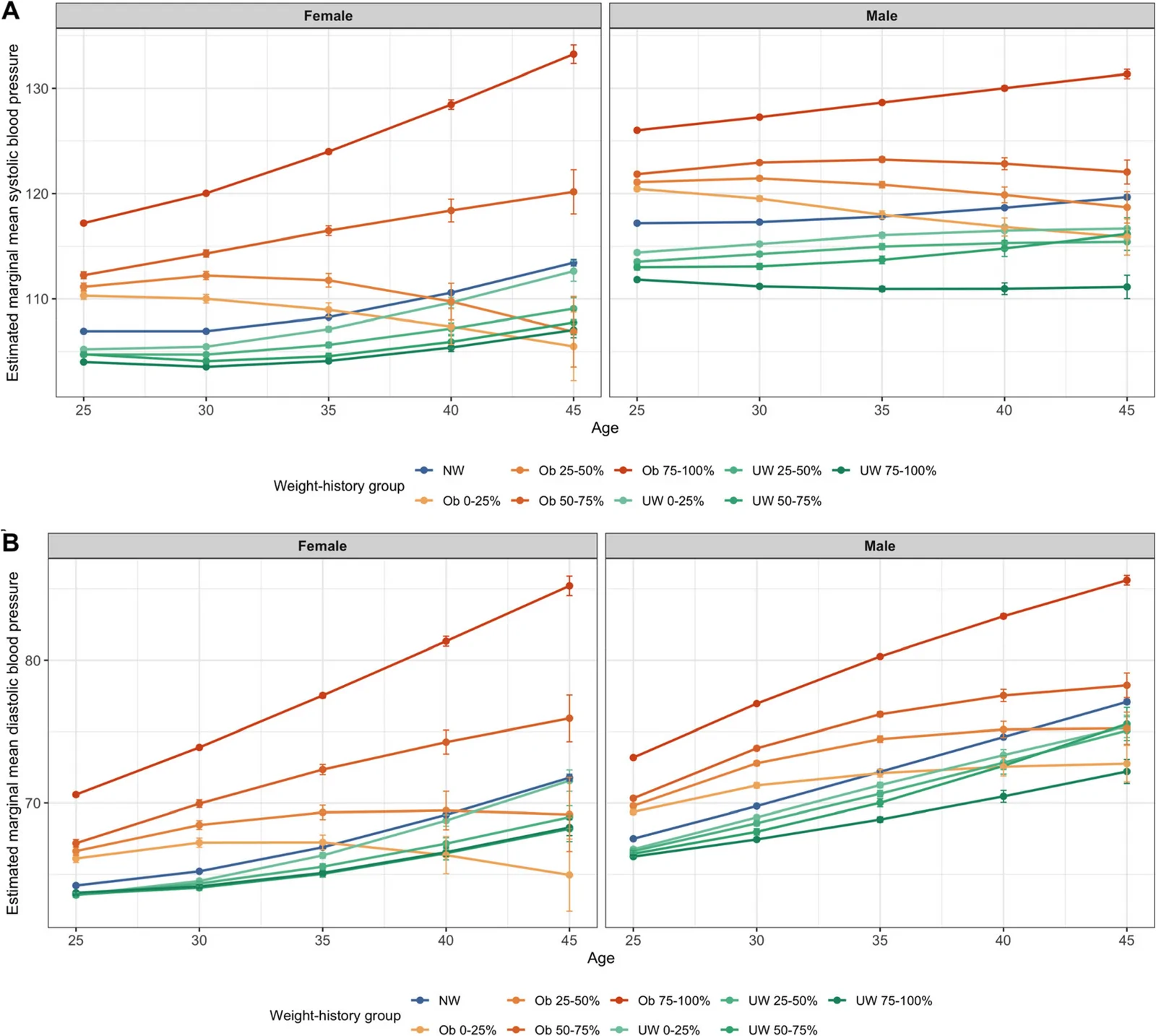
Nine-group blood pressure trajectories. Estimated marginal mean systolic blood pressure (SBP; panel **A**) and diastolic blood pressure (DBP; panel **B**) trajectories derived from sex-stratified linear mixed-effects models are shown for the nine weight-history groups. In each panel, results are shown separately for women and men. The underweight-history and obesity-history groups were further subdivided according to the proportion of observed follow-up time spent underweight or obese (0–25%, 25–50%, 50–75%, and 75–100%), while the normal-weight-only group was retained as the reference group. Models were adjusted for sleep, exercise habits, physical activity, and walking speed. Error bars indicate 95% confidence intervals. Abbreviations: NW, normal weight only throughout the 20s; Ob, obesity; UW, underweight

**Fig. 4:**
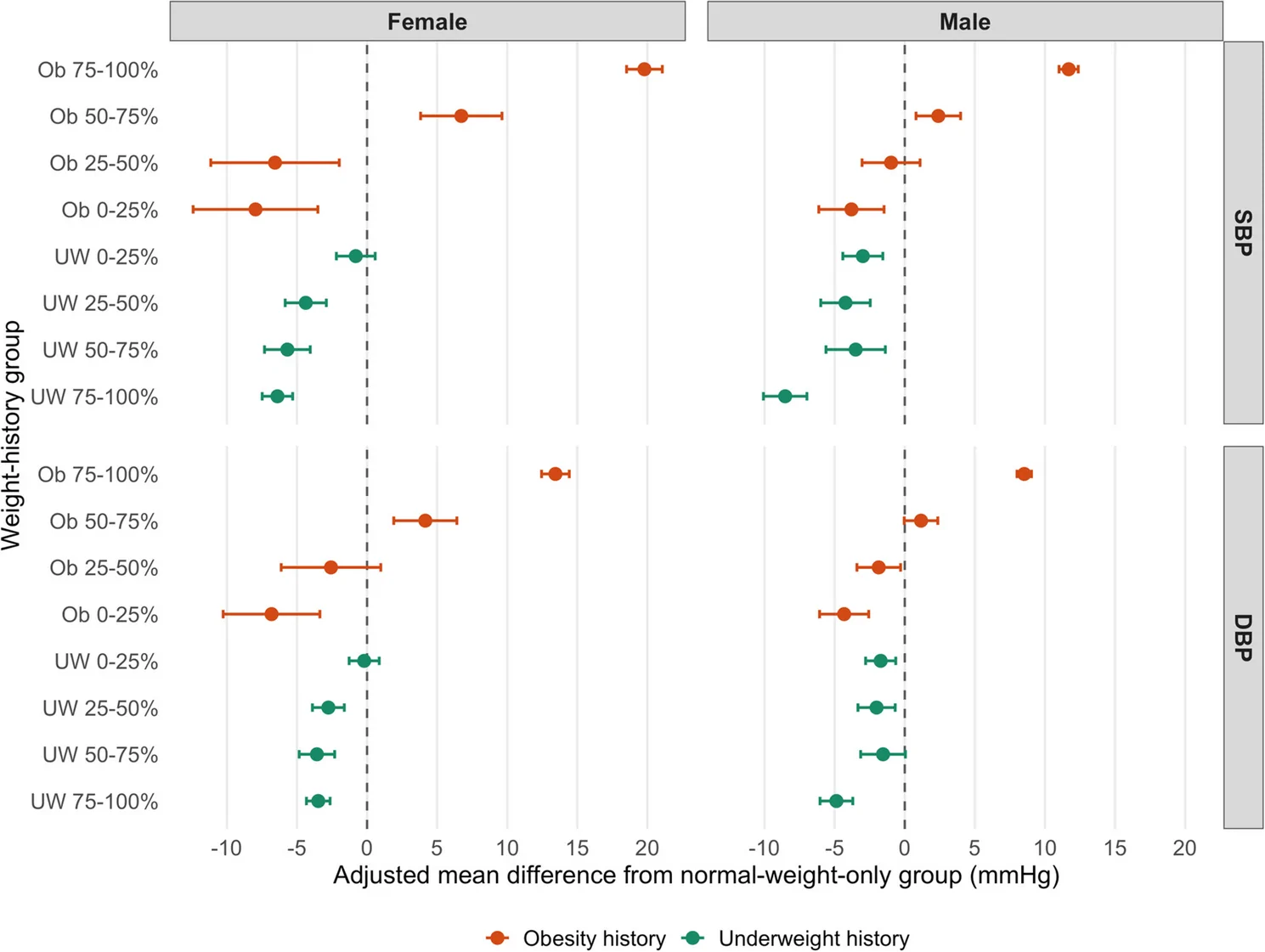
Age-45 blood pressure differences across nine weight-history groups. Adjusted mean differences in systolic blood pressure (SBP) and diastolic blood pressure (DBP) at age 45 years are shown relative to the normal-weight-only group for women and men. Points indicate adjusted mean differences and horizontal error bars indicate 95% confidence intervals. Negative values indicate lower blood pressure and positive values indicate higher blood pressure than the normal-weight-only group. The underweight-history and obesity-history groups were subdivided according to the proportion of observed follow-up time spent underweight or obese. Models were adjusted for sleep, exercise habits, physical activity, and walking speed. Abbreviations: UW, underweight; Ob, obesity; SBP, systolic blood pressure; DBP, diastolic blood pressure

Likelihood ratio tests also showed significant age-by-group interactions in the nine-group models for both women and men (all *P* < 0.001), indicating that age-related changes in SBP and DBP differed according to the more detailed weight-history classification. In the nine-group analysis, the Ob 75–100% group showed the highest SBP and DBP trajectories in both sexes, followed by the Ob 50–75% group. Differences between the Ob 0–25% and Ob 25–50% groups and the normal-weight-only group were smaller. Among the underweight-history subgroups, SBP and DBP trajectories were generally slightly lower than those in the normal-weight-only group (Fig. 3A and B and Supplementary Table 2). At age 45 years, the adjusted SBP/DBP differences relative to the normal-weight-only group were − 6.4/-3.5 mmHg in women and − 8.5/-4.9 mmHg in men in the UW 75–100% group, and + 19.8/+13.4 mmHg in women and + 11.7/+8.5 mmHg in men in the Ob 75–100% group (Fig. 4 and Supplementary Table 3).

## Discussion

In this study, we examined the association between body weight history during the 20s and subsequent blood pressure trajectories, as well as whether this association varied with age and was influenced by baseline eating behaviors. Participants with a history of obesity during their 20s showed consistently higher blood pressure than those who maintained normal weight throughout their 20s, and this difference became greater with increasing age. In contrast, participants with a history of underweight showed consistently lower blood pressure than the normal-weight-only group, and this difference also widened with age. Additional adjustment for baseline eating behaviors in sensitivity analyses did not materially alter these associations. Furthermore, when the obesity-history group was subdivided according to the proportion of follow-up time spent in obesity, participants in the 50–75% and 75–100% obesity-history groups had consistently higher blood pressure at each age than the normal-weight-only group, whereas participants in the 50–75% and 75–100% underweight-history groups had consistently lower blood pressure. These findings suggest that a longer duration of obesity or underweight is associated with persistently higher or lower blood pressure trajectories, respectively, compared with maintenance of normal weight.

Our findings revealed that the greater the proportion of time spent in obesity during the observation period, the higher the blood pressure, whereas a greater proportion of time spent underweight was associated with lower blood pressure. In recent years, cumulative exposure to obesity, which combines the degree and duration of obesity, has been increasingly evaluated using the concept of obesity [15–17]. Previous studies using this framework have shown that not only current BMI but also the duration of past obesity itself is associated with future hypertension and cardiovascular risk [15–17]. These observations are consistent with our finding that a longer obesity duration is associated with higher blood pressure. Therefore, the effect of obesity on blood pressure cannot be fully captured by a single cross-sectional measure of body size but should instead be understood as cumulative exposure over time.

A recent study of 56,459 Chinese adults reported progressively higher SBP, DBP, and odds of hypertension across weight-change patterns from young to middle adulthood, with the highest risk observed among participants with stable obesity [3]. These findings further support the importance of evaluating longitudinal body-weight patterns rather than body weight at a single time point. However, that study focused primarily on obesity-related weight transitions and BP measured in middle adulthood, whereas the present study additionally characterized repeated age-related BP trajectories associated with both obesity and underweight histories.

In contrast, although participants with a history of being underweight consistently had lower blood pressure, this should not be interpreted straightforwardly as indicating a more favorable cardiovascular profile. Previous studies have suggested that, compared with normal-weight individuals, underweight individuals may have a higher risk of adverse cardiovascular and mortality outcomes [18–22]. Thus, lower blood pressure in the underweight group does not necessarily imply a better overall prognosis. In addition, in our cohort, approximately 40% of individuals who were underweight during their 20s remained underweight thereafter, suggesting that underweight status was often persistent rather than transient [12, 13]. While our results indicate that a history of underweight is associated with lower blood pressure trajectories, the broader health implications of persistent underweight, including cardiovascular events, mortality, and frailty-related outcomes, require further investigation.

Among the dietary behaviors examined, eating speed was the most consistently significant factor. Previous studies have also reported an association between eating speed and blood pressure. Large-scale systematic reviews and meta-analyses have shown that, compared with slow eating, fast eating is associated with an increased risk of hypertension [9, 23]. A meta-analysis has also suggested that breakfast skipping is associated with a higher risk of hypertension [10]. However, in the present study, the blood pressure differences associated with eating behaviors were clearly smaller than those associated with the nine weight-history groups. Thus, although eating behaviors may act as modest modifiers of blood pressure, their contribution appears limited relative to the blood pressure burden associated with long-term body weight history.

Eating behaviors may change over time, and the baseline responses used in this study should not be interpreted as lifelong exposures. However, in our previous longitudinal analysis using the same health-check database, 72.2% of participants remained in the same self-reported eating-speed category at their final health checkup [12], suggesting that perceived eating speed was relatively stable for most participants during follow-up. Nevertheless, this stability was not complete and cannot be assumed for breakfast skipping or late-evening meal consumption.

Modest differences in baseline sleep and activity characteristics were observed across the weight-history groups, indicating their potential to confound the association between body-weight history and BP. The reported BP trajectories were therefore adjusted for these factors. However, because these variables were included as adjustment covariates rather than as primary exposures, their individual coefficients were not interpreted as independent or causal associations with BP.

The large sample size enabled precise estimation of potentially subtle differences in BP trajectories within a predominantly normotensive population, while also increasing the possibility that associations of limited magnitude would achieve statistical significance. We therefore interpreted the findings based not on P values alone but on the magnitude and precision of age-specific differences in mmHg, their consistency across SBP and DBP and both sexes, and their graded relationships with the duration of obesity or underweight. Although some contrasts were modest, the differences observed at older ages, particularly among participants with persistent obesity, were substantial.

The increasing separation of BP trajectories with age may reflect the superimposition of body-weight-related influences on the expected age-related increase in BP. Age-related BP elevation may involve progressive large-artery stiffening and vascular remodeling [24]. Experimental studies have also implicated reduced Klotho signaling with increased salt sensitivity [25] and age-dependent enhancement of angiotensin II signaling through AT1R-P2Y6R heterodimer formation [26]. In parallel, weight gain may contribute to higher BP through increased hemodynamic and renal mechanical load, sympathetic nervous system activation associated with metabolic and neurohumoral changes, and adipose-tissue-associated activation of the renin-angiotensin-aldosterone system [27]. These processes may provide biological context for the increasing separation of BP trajectories and the duration-related gradient observed in the obesity-history groups.

Several mechanisms might also contribute to the lower BP trajectories observed in the underweight-history groups. Smaller body and lean mass may be accompanied by lower circulating blood volume, stroke volume, and cardiac output. In some individuals, reduced skeletal-muscle mass or function may impair the lower-limb muscle pump and venous return, particularly during standing, while inadequate fluid or sodium intake and altered autonomic responses may further contribute to lower BP [28]. However, these factors were not measured, and dehydration, sodium insufficiency, or reduced sympathetic activity should not be assumed to characterize all individuals with an underweight history.

Moreover, visceral adiposity, hepatic steatosis, insulin resistance, vascular stiffness, circulating blood volume, autonomic function, and skeletal-muscle function were not directly assessed in the present study. Therefore, these considerations provide possible biological context but should not be interpreted as evidence of a specific causal or mediating mechanism. The lower BP trajectories observed in participants with an underweight history should likewise be interpreted as an epidemiological association rather than evidence of a favorable cardiovascular condition.

This study had several limitations. First, this observational study was based on routine health-check data from beneficiaries of an employment-based health insurance system, including employees and their dependents. Detailed information on socioeconomic and social backgrounds was unavailable. In addition, individuals who underwent repeated health checkups may differ from the general population in employment status, access to health care, and health-related behaviors. Therefore, the findings may not be directly generalizable to the overall Japanese population, individuals outside employment-based insurance systems, populations without regular health checkups, older adults, or patients with established hypertension.

Second, eating behaviors, including eating speed, breakfast skipping, and late-evening meals, as well as sleep adequacy, exercise habits, physical activity, and walking speed, were assessed using self-reported questionnaire responses. These variables may therefore have been affected by recall bias, reporting bias, and exposure misclassification. In addition, smoking, alcohol consumption, socioeconomic status, occupational category, and marital status were not incorporated into the analysis; therefore, residual confounding cannot be excluded.

Third, this study relied entirely on secondary data collected during routine health checkups. Detailed information on measurement protocols, device calibration, quality-assurance procedures, and the consistency of anthropometric and BP measurements across health-check centers was unavailable. In particular, the database did not identify whether BP was measured manually or with an automated device. Therefore, we could not distinguish potential inter-observer variability in manual measurements from variability related to devices or settings in automated measurements. Although these measurements were expected to follow routine health-check procedures, differences in devices, examiners, timing, and participant conditions may have introduced measurement error and between-center variability. Large-scale database studies such as the present study provide population heterogeneity and sufficient precision to characterize age-specific trajectories, but they offer less control over measurement conditions than smaller, intensively characterized cohorts. Conversely, smaller cohorts allow greater measurement standardization but may be more susceptible to selection and center-specific effects. These study designs should therefore be viewed as complementary, and the large sample size does not eliminate systematic measurement bias or residual confounding. Ideally, the present findings should be validated in a prospective multicenter study using standardized measurement protocols, calibrated devices, and centralized quality-control procedures. However, even under such a design, complete adherence to measurement protocols cannot be guaranteed, and some inter-center and examiner-related variability may remain. Nevertheless, the magnitude of the observed differences, their consistency across SBP and DBP and both sexes, and the graded patterns according to the duration of obesity or underweight suggest that the findings are unlikely to be explained entirely by random measurement variability.

Fourth, eating behaviors may have changed in response to weight gain or BP elevation, and reverse-direction relationships are therefore plausible. Causality cannot be established from the present observational design. To preserve temporal ordering, we used eating behaviors reported at the first health checkup in participants’ 20s as baseline variables. In our previous longitudinal analysis using the same health-check database, 72.2% of participants remained in the same self-reported eating-speed category at their final health checkup [12], indicating relative, but not complete, stability during follow-up. However, the longitudinal stability of breakfast skipping and late-evening meal consumption was not established. Future studies incorporating repeated assessments and time-varying exposure models would therefore be informative. Fifth, because multiple secondary interactions and subgroup-specific contrast analyses were performed, some nominally significant small effects may reflect chance findings. These results should therefore be interpreted as exploratory, with greater emphasis on the overall magnitude and consistency of associations than on individual nominal P values.

Sixth, although the follow-up duration and number of health checkups were broadly similar across weight-history groups, the timing and frequency of repeated health checkups still varied across participants, and differential loss to follow-up related to both body weight history and blood pressure cannot be excluded. Thus, bias due to informative missingness is possible.

Seventh, information on a history of hypertension and antihypertensive medication use was not incorporated into the analysis. However, the 2023 National Health and Nutrition Survey of Japan reported antihypertensive medication use in 0.0%, 2.5%, and 6.6% of adults aged 20–29, 30–39, and 40–49 years, respectively [29]. Given the similarly young age and predominantly normotensive BP distribution of our study population, the influence of antihypertensive treatment on the overall findings was therefore likely limited. Any remaining treatment effect at older ages, particularly among participants with a history of obesity, would be expected to lower observed BP and thus modestly attenuate rather than exaggerate the estimated between-group differences.

Finally, the study population consisted of employment-based health insurance beneficiaries who underwent repeated health checkups and was not representative of the general Japanese population. The findings may therefore not be fully generalizable to individuals outside employment-based insurance systems, those without regular health checkups, populations with established hypertension, or other demographic settings. In addition, because the present analysis evaluated age-related trajectories within an observational cohort, the observed patterns may partly reflect cohort or period effects rather than pure age effects alone.

## Conclusion

Body weight history was a stronger determinant of blood pressure burden than baseline eating behaviors. A longer duration of obesity was associated with persistently higher blood pressure trajectories, whereas participants with a shorter duration of obesity had blood pressure levels closer to those of the normal-weight-only group, even into their 40s. Conversely, participants with a longer duration of underweight showed consistently lower blood pressure trajectories and appeared less affected by age-related increases in blood pressure. These findings suggest that minimizing the duration of obesity and underweight may be associated with blood pressure trajectories closer to those of the normal-weight-only group. More broadly, when evaluating later blood pressure trajectories, greater attention should be paid to body weight history across early adulthood rather than to body weight at a single time point.

## Supplementary Information

Supplementary Material 1.

## Data Availability

The data that support the findings of this study were obtained from JMDC Inc. and are not publicly available because of contractual and privacy restrictions. Data may be available from the corresponding author upon reasonable request and with permission from JMDC Inc.

## Abbreviations

BMI: Body mass index
BP: Blood pressure
SBP: Systolic blood pressure
DBP: Diastolic blood pressure
UW: Underweight
NW: Normal weight
Ob: Obesity
