# Supplementary material for: Body weight history from young adulthood and subsequent blood pressure trajectories in a Japanese employment-based health-check cohort: a retrospective longitudinal study
Source: BMC Public Health. 2026 Aug 19;26:3374. doi: 10.1186/s12889-026-29097-z (PMC13760771; doi:10.1186/s12889-026-29097-z)
Supplement: Supplementary file 1 [file 12889_2026_29097_MOESM1_ESM.docx]

***Supplementary Table 1. Estimated marginal means of blood pressure at ages 25, 35, and 45 years in the three-group analysis***

| **Outcome** | **Sex** | **Weight-history group** | **Age 25 years** | | **Age 35 years** | | **Age 45 years** | |
| --- | --- | --- | --- | --- | --- | --- | --- | --- |
|  |  |  | **Adjusted** | **Additively adjusted** | **Adjusted** | **Additively adjusted** | **Adjusted** | **Additively adjusted** |
| SBP | Female | History of underweight | 104.497 [104.406, 104.588] | 104.164 [104.044, 104.283] | 105.096 [104.977, 105.215] | 104.741 [104.600, 104.883] | 108.830 [108.364, 109.296] | 108.480 [108.002, 108.958] |
| SBP | Female | Normal weight only in 20s | 106.933 [106.863, 107.004] | 106.555 [106.454, 106.657] | 108.272 [108.189, 108.354] | 107.891 [107.781, 108.002] | 113.447 [113.132, 113.761] | 113.009 [112.683, 113.335] |
| SBP | Female | History of obesity | 114.646 [114.509, 114.783] | 114.258 [114.103, 114.412] | 120.338 [120.161, 120.515] | 119.980 [119.788, 120.171] | 128.461 [127.700, 129.222] | 128.114 [127.341, 128.886] |
| SBP | Male | History of underweight | 113.029 [112.924, 113.133] | 112.932 [112.820, 113.044] | 113.697 [113.558, 113.835] | 113.592 [113.447, 113.736] | 114.583 [113.985, 115.181] | 114.464 [113.861, 115.067] |
| SBP | Male | Normal weight only in 20s | 117.186 [117.132, 117.239] | 116.908 [116.842, 116.975] | 117.817 [117.758, 117.877] | 117.529 [117.458, 117.601] | 119.660 [119.431, 119.889] | 119.387 [119.153, 119.621] |
| SBP | Male | History of obesity | 124.152 [124.080, 124.224] | 123.739 [123.655, 123.823] | 126.357 [126.269, 126.446] | 125.936 [125.838, 126.035] | 128.484 [128.095, 128.872] | 128.033 [127.640, 128.426] |
| DBP | Female | History of underweight | 63.632 [63.561, 63.703] | 63.360 [63.267, 63.454] | 65.443 [65.351, 65.536] | 65.166 [65.056, 65.277] | 69.181 [68.820, 69.542] | 68.952 [68.581, 69.322] |
| DBP | Female | Normal weight only in 20s | 64.218 [64.163, 64.273] | 63.933 [63.854, 64.013] | 66.906 [66.841, 66.970] | 66.629 [66.543, 66.716] | 71.776 [71.533, 72.020] | 71.485 [71.232, 71.738] |
| DBP | Female | History of obesity | 68.915 [68.808, 69.022] | 68.628 [68.507, 68.749] | 75.044 [74.906, 75.182] | 74.758 [74.609, 74.907] | 81.942 [81.352, 82.532] | 81.624 [81.025, 82.223] |
| DBP | Male | History of underweight | 66.481 [66.399, 66.563] | 66.244 [66.156, 66.332] | 70.140 [70.033, 70.247] | 69.900 [69.788, 70.012] | 74.397 [73.947, 74.847] | 74.134 [73.680, 74.588] |
| DBP | Male | Normal weight only in 20s | 67.506 [67.465, 67.548] | 67.183 [67.131, 67.235] | 72.186 [72.139, 72.232] | 71.856 [71.800, 71.912] | 77.083 [76.910, 77.256] | 76.768 [76.592, 76.945] |
| DBP | Male | History of obesity | 71.938 [71.881, 71.994] | 71.534 [71.468, 71.600] | 78.546 [78.478, 78.614] | 78.131 [78.055, 78.208] | 83.275 [82.983, 83.568] | 82.855 [82.559, 83.151] |

Values are shown as mean [95% CI]. Adjusted models were adjusted for sleep, exercise habits, physical activity, and walking speed. Additionally adjusted models included eating speed, breakfast skipping, and late-evening meal consumption. CI, confidence interval; SBP, systolic blood pressure; DBP, diastolic blood pressure.

***Supplementary Table 2. Estimated marginal means of blood pressure at ages 25, 35, and 45 years in nine weight-history groups***

|  | | | **Age 25 years** | **Age 35 years** | **Age 45 years** |
| --- | --- | --- | --- | --- | --- |
|  | | | **Adjusted** | **Adjusted** | **Adjusted** |
| SBP | Female | UW 0-25% | 104.86 [104.64, 105.07] | 106.78 [106.51, 107.06] | 112.44 [111.46, 113.41] |
| SBP | Female | UW 25-50% | 104.37 [104.16, 104.59] | 105.25 [104.97, 105.53] | 108.59 [107.54, 109.64] |
| SBP | Female | UW 50-75% | 104.38 [104.14, 104.62] | 104.20 [103.89, 104.50] | 107.47 [106.30, 108.64] |
| SBP | Female | UW 75-100% | 103.68 [103.52, 103.83] | 103.73 [103.54, 103.93] | 106.62 [105.87, 107.37] |
| SBP | Female | NW | 106.55 [106.45, 106.65] | 107.89 [107.78, 108.00] | 113.01 [112.68, 113.33] |
| SBP | Female | Ob 0-25% | 109.95 [109.59, 110.31] | 108.60 [107.94, 109.27] | 105.49 [102.19, 108.79] |
| SBP | Female | Ob 25-50% | 110.77 [110.41, 111.14] | 111.38 [110.72, 112.05] | 106.39 [102.94, 109.83] |
| SBP | Female | Ob 50-75% | 111.89 [111.55, 112.24] | 116.08 [115.62, 116.55] | 119.74 [117.62, 121.87] |
| SBP | Female | Ob 75-100% | 116.79 [116.59, 116.99] | 123.62 [123.40, 123.85] | 132.81 [131.91, 133.70] |
| SBP | Male | UW 0-25% | 114.26 [114.05, 114.46] | 115.88 [115.63, 116.14] | 116.37 [115.34, 117.40] |
| SBP | Male | UW 25-50% | 113.39 [113.16, 113.61] | 114.83 [114.53, 115.13] | 115.26 [113.97, 116.55] |
| SBP | Male | UW 50-75% | 112.90 [112.62, 113.18] | 113.61 [113.25, 113.98] | 116.11 [114.57, 117.65] |
| SBP | Male | UW 75-100% | 111.76 [111.59, 111.93] | 110.86 [110.61, 111.10] | 111.17 [110.06, 112.29] |
| SBP | Male | NW | 116.91 [116.85, 116.98] | 117.54 [117.47, 117.61] | 119.40 [119.17, 119.63] |
| SBP | Male | Ob 0-25% | 120.13 [119.91, 120.36] | 117.64 [117.27, 118.00] | 115.47 [113.77, 117.18] |
| SBP | Male | Ob 25-50% | 120.76 [120.55, 120.96] | 120.53 [120.23, 120.83] | 118.22 [116.71, 119.73] |
| SBP | Male | Ob 50-75% | 121.51 [121.33, 121.69] | 122.88 [122.65, 123.12] | 121.75 [120.60, 122.90] |
| SBP | Male | Ob 75-100% | 125.60 [125.50, 125.71] | 128.25 [128.13, 128.36] | 130.93 [130.48, 131.38] |
| DBP | Female | UW 0-25% | 63.28 [63.11, 63.45] | 66.07 [65.86, 66.28] | 71.41 [70.65, 72.17] |
| DBP | Female | UW 25-50% | 63.31 [63.14, 63.48] | 65.26 [65.04, 65.48] | 68.72 [67.90, 69.53] |
| DBP | Female | UW 50-75% | 63.33 [63.14, 63.52] | 64.78 [64.54, 65.02] | 68.01 [67.10, 68.92] |
| DBP | Female | UW 75-100% | 63.43 [63.31, 63.55] | 64.80 [64.65, 64.95] | 68.01 [67.43, 68.59] |
| DBP | Female | NW | 63.93 [63.85, 64.01] | 66.63 [66.54, 66.71] | 71.48 [71.23, 71.73] |
| DBP | Female | Ob 0-25% | 65.85 [65.56, 66.14] | 66.93 [66.42, 67.45] | 64.57 [62.01, 67.12] |
| DBP | Female | Ob 25-50% | 66.35 [66.06, 66.64] | 68.92 [68.41, 69.44] | 68.21 [65.54, 70.88] |
| DBP | Female | Ob 50-75% | 66.91 [66.64, 67.18] | 72.06 [71.69, 72.42] | 75.66 [74.01, 77.31] |
| DBP | Female | Ob 75-100% | 70.30 [70.14, 70.45] | 77.25 [77.08, 77.43] | 84.88 [84.19, 85.57] |
| DBP | Male | UW 0-25% | 66.49 [66.33, 66.65] | 70.98 [70.78, 71.18] | 75.03 [74.25, 75.80] |
| DBP | Male | UW 25-50% | 66.36 [66.18, 66.53] | 70.39 [70.16, 70.62] | 74.77 [73.80, 75.75] |
| DBP | Male | UW 50-75% | 66.21 [65.99, 66.43] | 69.77 [69.48, 70.05] | 75.29 [74.13, 76.45] |
| DBP | Male | UW 75-100% | 66.02 [65.88, 66.15] | 68.61 [68.42, 68.80] | 72.00 [71.16, 72.84] |
| DBP | Male | NW | 67.18 [67.13, 67.23] | 71.86 [71.80, 71.91] | 76.77 [76.60, 76.95] |
| DBP | Male | Ob 0-25% | 69.03 [68.85, 69.20] | 71.71 [71.42, 71.99] | 72.32 [71.03, 73.60] |
| DBP | Male | Ob 25-50% | 69.45 [69.29, 69.61] | 74.10 [73.87, 74.34] | 74.73 [73.59, 75.86] |
| DBP | Male | Ob 50-75% | 69.96 [69.82, 70.11] | 75.82 [75.64, 76.00] | 77.92 [77.05, 78.78] |
| DBP | Male | Ob 75-100% | 72.79 [72.71, 72.87] | 79.86 [79.77, 79.95] | 85.21 [84.86, 85.55] |

Values are shown as mean [95% CI]. Models were adjusted for sleep, exercise habits, physical activity, and walking speed. CI, confidence interval; SBP, systolic blood pressure; DBP, diastolic blood pressure; UW, underweight; NW, normal weight only throughout the 20s; Ob, obesity.

Supplementary Table 3. Adjusted blood pressure differences from normal weight at age 45 years

| **Classification** | **Comparison** | **Sex** | **SBP difference** | **DBP difference** |
| --- | --- | --- | --- | --- |
| Three-group | History of UW | Female | -4.6 [-5.3, -4.0] | -2.6 [-3.1, -2.1] |
| Three-group | History of UW | Male | -5.1 [-5.8, -4.4] | -2.7 [-3.2, -2.1] |
| Three-group | History of Ob | Female | +15.0 [14.1, 15.9] | +10.2 [9.4, 10.9] |
| Three-group | History of Ob | Male | +8.8 [8.3, 9.3] | +6.2 [5.8, 6.6] |
| Nine-group | UW 0-25% | Female | -0.8 [-2.2, 0.6] | -0.2 [-1.3, 0.9] |
| Nine-group | UW 0-25% | Male | -3.0 [-4.4, -1.6] | -1.7 [-2.8, -0.6] |
| Nine-group | UW 25-50% | Female | -4.4 [-5.8, -2.9] | -2.8 [-3.9, -1.6] |
| Nine-group | UW 25-50% | Male | -4.2 [-6.0, -2.5] | -2.0 [-3.3, -0.7] |
| Nine-group | UW 50-75% | Female | -5.7 [-7.3, -4.1] | -3.6 [-4.8, -2.3] |
| Nine-group | UW 50-75% | Male | -3.5 [-5.6, -1.4] | -1.5 [-3.1, 0.0] |
| Nine-group | UW 75-100% | Female | -6.4 [-7.5, -5.3] | -3.5 [-4.3, -2.6] |
| Nine-group | UW 75-100% | Male | -8.5 [-10.1, -7.0] | -4.9 [-6.0, -3.7] |
| Nine-group | Ob 0-25% | Female | -8.0 [-12.4, -3.5] | -6.8 [-10.3, -3.4] |
| Nine-group | Ob 0-25% | Male | -3.8 [-6.1, -1.5] | -4.3 [-6.1, -2.6] |
| Nine-group | Ob 25-50% | Female | -6.6 [-11.1, -2.0] | -2.6 [-6.1, 1.0] |
| Nine-group | Ob 25-50% | Male | -1.0 [-3.0, 1.1] | -1.9 [-3.4, -0.3] |
| Nine-group | Ob 50-75% | Female | +6.7 [3.8, 9.6] | +4.2 [1.9, 6.4] |
| Nine-group | Ob 50-75% | Male | +2.4 [0.8, 4.0] | +1.2 [0.0, 2.4] |
| Nine-group | Ob 75-100% | Female | +19.8 [18.5, 21.1] | +13.4 [12.4, 14.4] |
| Nine-group | Ob 75-100% | Male | +11.7 [11.0, 12.4] | +8.5 [8.0, 9.0] |

Values are adjusted mean differences [95% CI] in mmHg relative to the normal-weight-only group. Negative values indicate lower blood pressure and positive values indicate higher blood pressure. CI, confidence interval; SBP, systolic blood pressure; DBP, diastolic blood pressure; UW, underweight; Ob, obesity.
